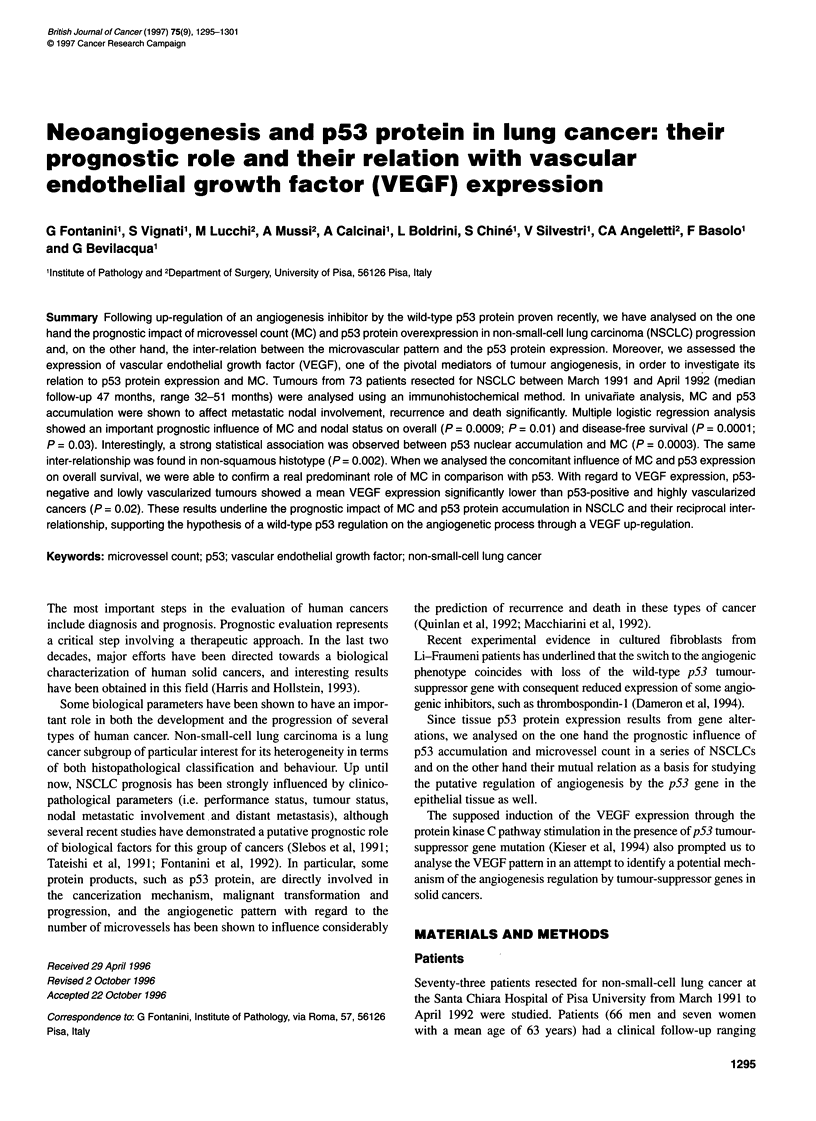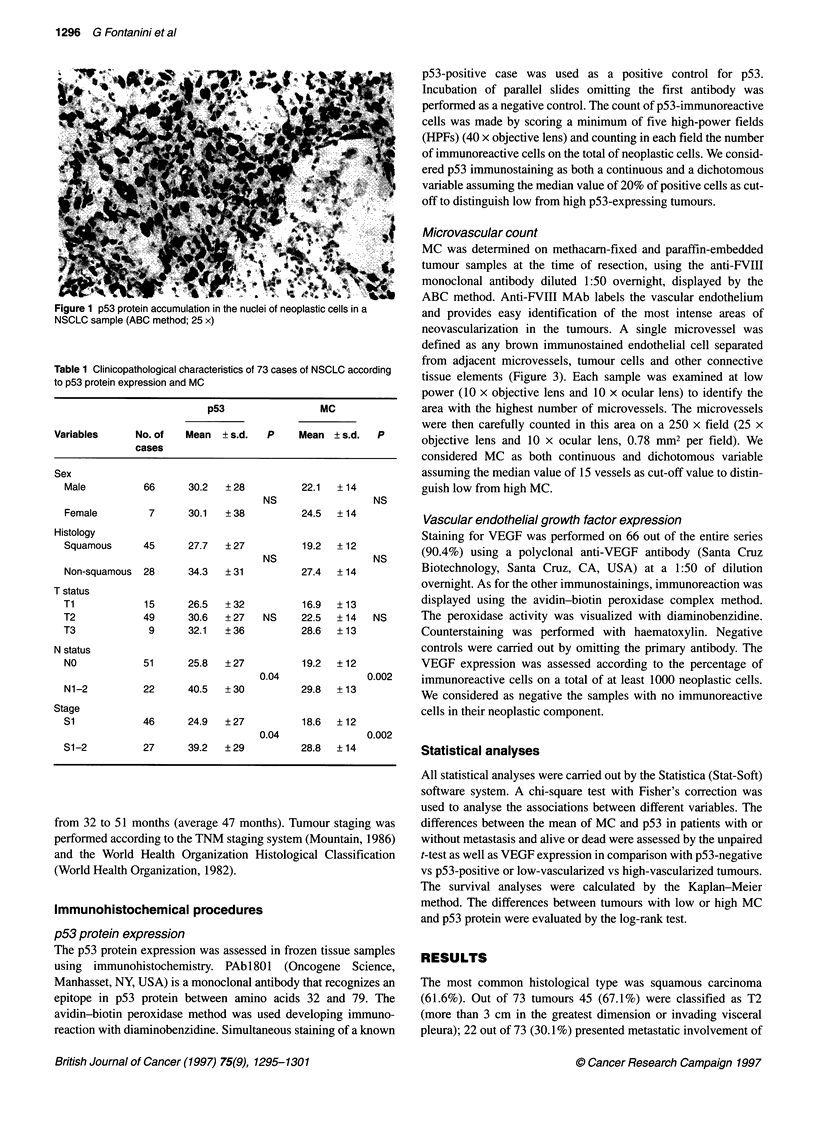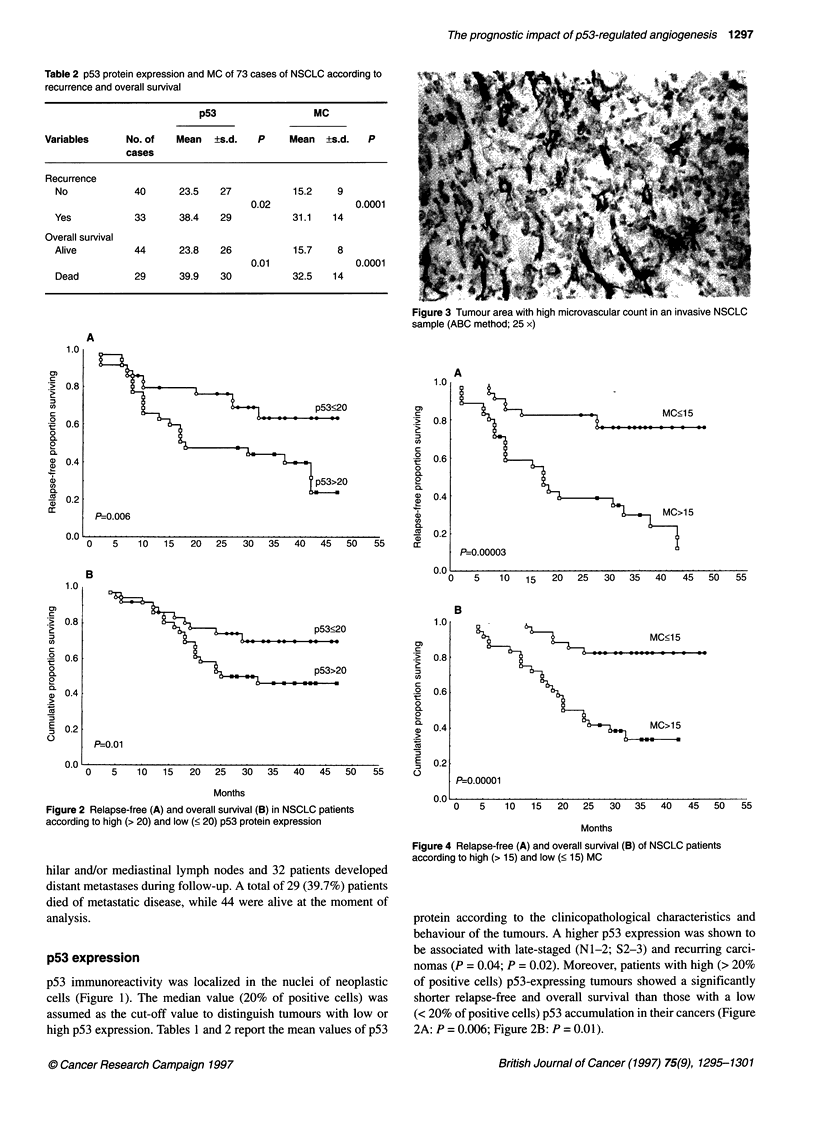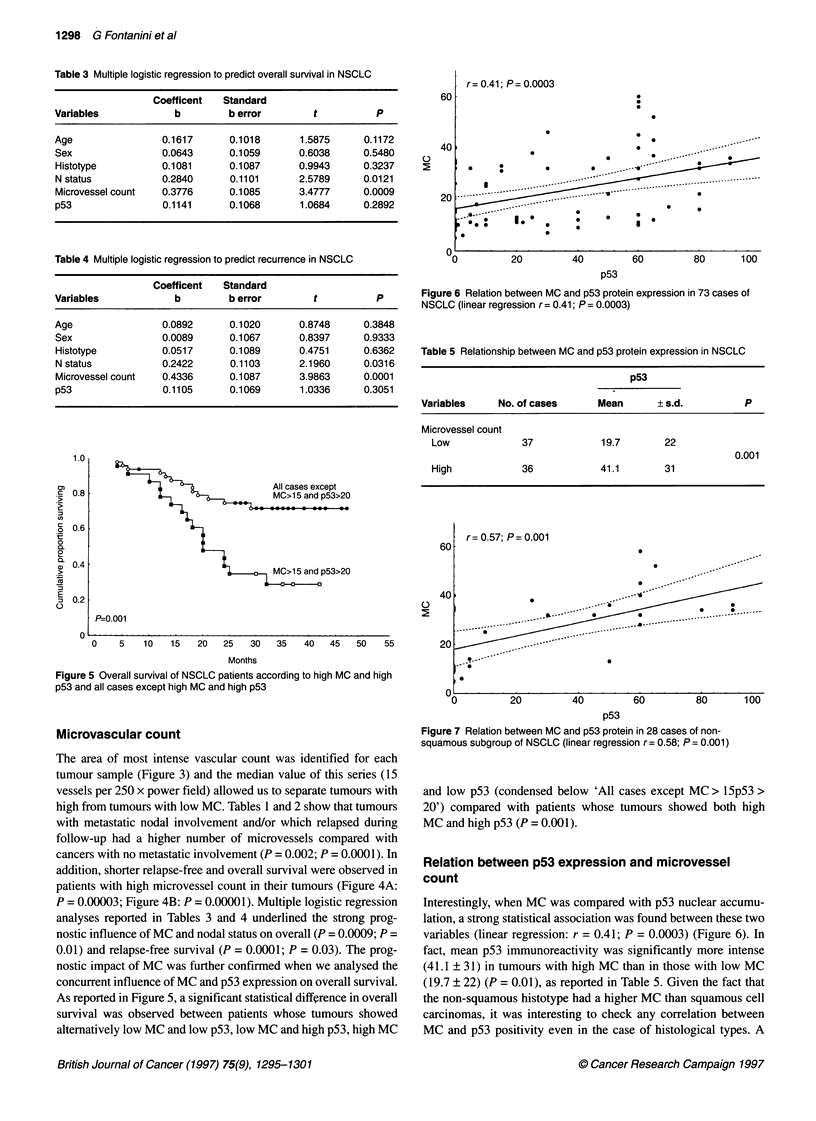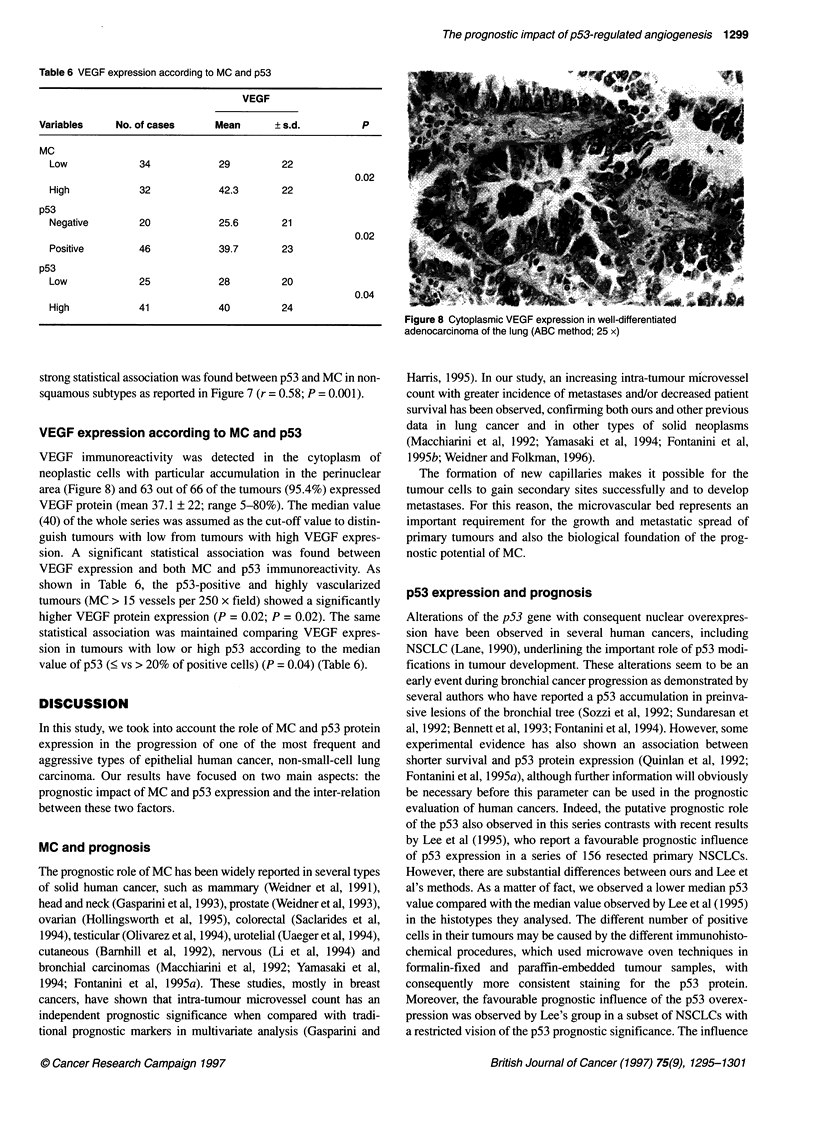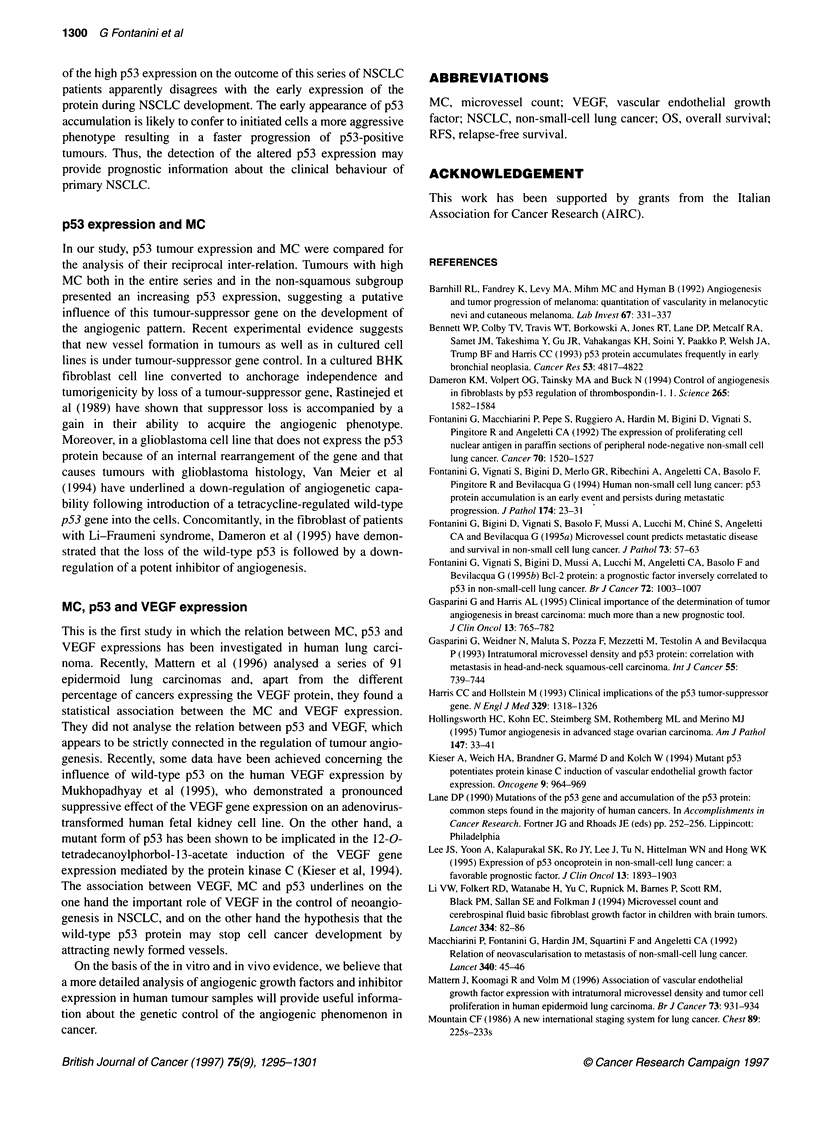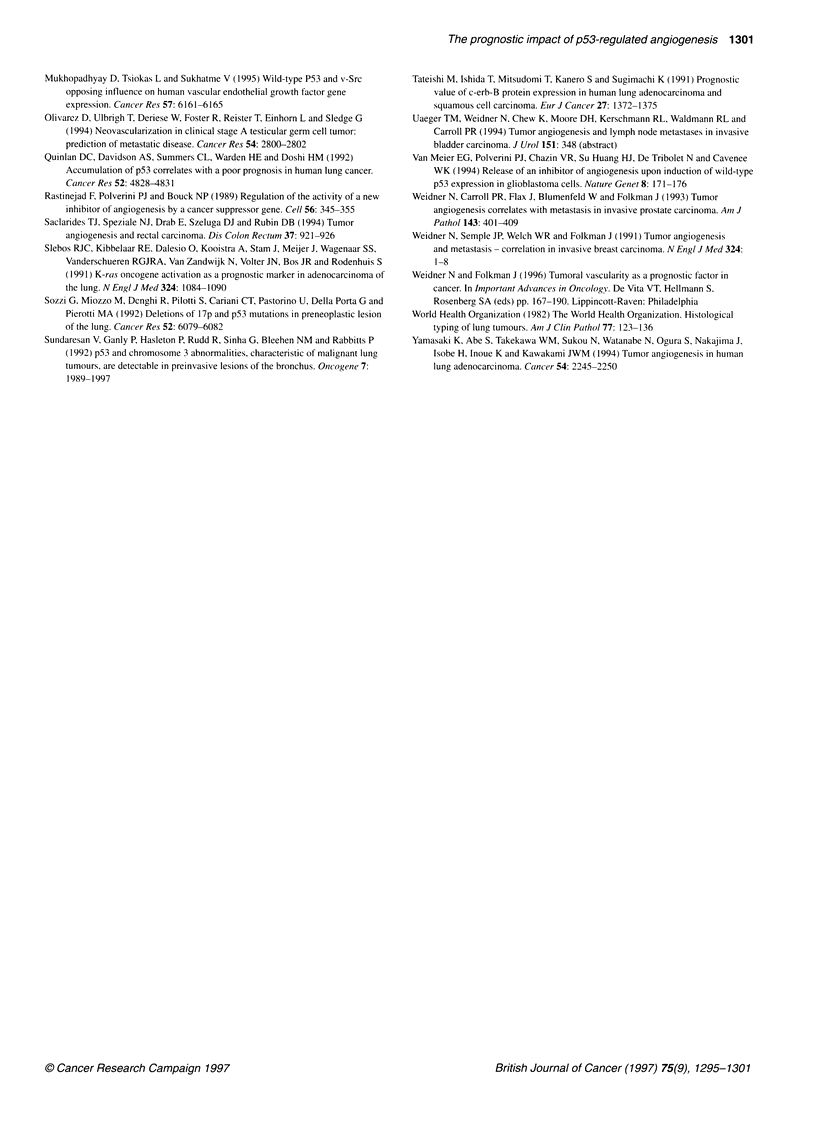# Neoangiogenesis and p53 protein in lung cancer: their prognostic role and their relation with vascular endothelial growth factor (VEGF) expression.

**DOI:** 10.1038/bjc.1997.220

**Published:** 1997

**Authors:** G. Fontanini, S. Vignati, M. Lucchi, A. Mussi, A. Calcinai, L. Boldrini, S. ChinÃ©, V. Silvestri, C. A. Angeletti, F. Basolo, G. Bevilacqua

**Affiliations:** Institute of Pathology, University of Pisa, Italy.

## Abstract

**Images:**


British Journal of Cancer (1997) 75(9), 1295–1301
© 1997 Cancer Research Campaign

### Neoangiogenesis and p53 protein in lung cancer: their prognostic role and their relation with vascular endothelial growth factor (VEGF) expression

**G Fontanini[1], S Vignati[1], M Lucchi[2], A Mussi[2], A Calcinai[1], L Boldrini, S Chiné[1], V Silvestri[1], CA Angeletti[2], F Basolo[1] and G Bevilacqua[1]**

[1]Institute of Pathology and [2]Department of Surgery, University of Pisa, 56126 Pisa, Italy

**Summary** Following up-regulation of an angiogenesis inhibitor by the wild-type p53 protein proven recently, we have analysed on the one hand the prognostic impact of microvessel count (MC) and p53 protein overexpression in non-small-cell lung carcinoma (NSCLC) progression and, on the other hand, the inter-relation between the microvascular pattern and the p53 protein expression. Moreover, we assessed the expression of vascular endothelial growth factor (VEGF), one of the pivotal mediators of tumour angiogenesis, in order to investigate its relation to p53 protein expression and MC. Tumours from 73 patients resected for NSCLC between March 1991 and April 1992 (median follow-up 47 months, range 32–51 months) were analysed using an immunohistochemical method. In univariate analysis, MC and p53 accumulation were shown to affect metastatic nodal involvement, recurrence and death significantly. Multiple logistic regression analysis showed an important prognostic influence of MC and nodal status on overall ($P = 0.0009$; $P = 0.01$) and disease-free survival ($P = 0.0001$; $P = 0.03$). Interestingly, a strong statistical association was observed between p53 nuclear accumulation and MC ($P = 0.0003$). The same inter-relationship was found in non-squamous histotype ($P = 0.002$). When we analysed the concomitant influence of MC and p53 expression on overall survival, we were able to confirm a real predominant role of MC in comparison with p53. With regard to VEGF expression, p53-negative and lowly vascularized tumours showed a mean VEGF expression significantly lower than p53-positive and highly vascularized cancers ($P = 0.02$). These results underline the prognostic impact of MC and p53 protein accumulation in NSCLC and their reciprocal inter-relationship, supporting the hypothesis of a wild-type p53 regulation on the angiogenetic process through a VEGF up-regulation.

**Keywords:** microvessel count; p53; vascular endothelial growth factor; non-small-cell lung cancer

The most important steps in the evaluation of human cancers include diagnosis and prognosis. Prognostic evaluation represents a critical step involving a therapeutic approach. In the last two decades, major efforts have been directed towards a biological characterization of human solid cancers, and interesting results have been obtained in this field (Harris and Hollstein, 1993).

Some biological parameters have been shown to have an important role in both the development and the progression of several types of human cancer. Non-small-cell lung carcinoma is a lung cancer subgroup of particular interest for its heterogeneity in terms of both histopathological classification and behaviour. Up until now, NSCLC prognosis has been strongly influenced by clinicopathological parameters (i.e. performance status, tumour status, nodal metastatic involvement and distant metastasis), although several recent studies have demonstrated a putative prognostic role of biological factors for this group of cancers (Slebos et al, 1991; Tateishi et al, 1991; Fontanini et al, 1992). In particular, some protein products, such as p53 protein, are directly involved in the cancerization mechanism, malignant transformation and progression, and the angiogenetic pattern with regard to the number of microvessels has been shown to influence considerably the prediction of recurrence and death in these types of cancer (Quinlan et al, 1992; Macchiarini et al, 1992).

*Received 29 April 1996*
*Revised 2 October 1996*
*Accepted 22 October 1996*

*Correspondence to*: G Fontanini, Institute of Pathology, via Roma, 57, 56126 Pisa, Italy

Recent experimental evidence in cultured fibroblasts from Li–Fraumeni patients has underlined that the switch to the angiogenic phenotype coincides with loss of the wild-type *p53* tumour-suppressor gene with consequent reduced expression of some angiogenic inhibitors, such as thrombospondin-1 (Dameron et al, 1994).

Since tissue p53 protein expression results from gene alterations, we analysed on the one hand the prognostic influence of p53 accumulation and microvessel count in a series of NSCLCs and on the other hand their mutual relation as a basis for studying the putative regulation of angiogenesis by the *p53* gene in the epithelial tissue as well.

The supposed induction of the VEGF expression through the protein kinase C pathway stimulation in the presence of *p53* tumour-suppressor gene mutation (Kieser et al, 1994) also prompted us to analyse the VEGF pattern in an attempt to identify a potential mechanism of the angiogenesis regulation by tumour-suppressor genes in solid cancers.

### MATERIALS AND METHODS

### Patients

Seventy-three patients resected for non-small-cell lung cancer at the Santa Chiara Hospital of Pisa University from March 1991 to April 1992 were studied. Patients (66 men and seven women with a mean age of 63 years) had a clinical follow-up ranging

**Figure 1** p53 protein accumulation in the nuclei of neoplastic cells in a NSCLC sample (ABC method; 25 ×)

**Table 1** Clinicopathological characteristics of 73 cases of NSCLC according to p53 protein expression and MC

| | | p53 | | | MC | | |
|---|---|---|---|---|---|---|---|
| **Variables** | **No. of cases** | **Mean** | **± s.d.** | ***P*** | **Mean** | **± s.d.** | ***P*** |
| Sex | | | | | | | |
| Male | 66 | 30.2 | ± 28 | | 22.1 | ± 14 | |
| | | | | NS | | | NS |
| Female | 7 | 30.1 | ± 38 | | 24.5 | ± 14 | |
| Histology | | | | | | | |
| Squamous | 45 | 27.7 | ± 27 | | 19.2 | ± 12 | |
| | | | | NS | | | NS |
| Non-squamous | 28 | 34.3 | ± 31 | | 27.4 | ± 14 | |
| T status | | | | | | | |
| T1 | 15 | 26.5 | ± 32 | | 16.9 | ± 13 | |
| T2 | 49 | 30.6 | ± 27 | NS | 22.5 | ± 14 | NS |
| T3 | 9 | 32.1 | ± 36 | | 28.6 | ± 13 | |
| N status | | | | | | | |
| N0 | 51 | 25.8 | ± 27 | | 19.2 | ± 12 | |
| | | | | 0.04 | | | 0.002 |
| N1–2 | 22 | 40.5 | ± 30 | | 29.8 | ± 13 | |
| Stage | | | | | | | |
| S1 | 46 | 24.9 | ± 27 | | 18.6 | ± 12 | |
| | | | | 0.04 | | | 0.002 |
| S1–2 | 27 | 39.2 | ± 29 | | 28.8 | ± 14 | |

from 32 to 51 months (average 47 months). Tumour staging was performed according to the TNM staging system (Mountain, 1986) and the World Health Organization Histological Classification (World Health Organization, 1982).

### Immunohistochemical procedures

### *p53 protein expression*

The p53 protein expression was assessed in frozen tissue samples using immunohistochemistry. PAb1801 (Oncogene Science, Manhasset, NY, USA) is a monoclonal antibody that recognizes an epitope in p53 protein between amino acids 32 and 79. The avidin–biotin peroxidase method was used developing immunoreaction with diaminobenzidine. Simultaneous staining of a known p53-positive case was used as a positive control for p53. Incubation of parallel slides omitting the first antibody was performed as a negative control. The count of p53-immunoreactive cells was made by scoring a minimum of five high-power fields (HPFs) (40 × objective lens) and counting in each field the number of immunoreactive cells on the total of neoplastic cells. We considered p53 immunostaining as both a continuous and a dichotomous variable assuming the median value of 20% of positive cells as cut-off to distinguish low from high p53-expressing tumours.

### *Microvascular count*

MC was determined on methacarn-fixed and paraffin-embedded tumour samples at the time of resection, using the anti-FVIII monoclonal antibody diluted 1:50 overnight, displayed by the ABC method. Anti-FVIII MAb labels the vascular endothelium and provides easy identification of the most intense areas of neovascularization in the tumours. A single microvessel was defined as any brown immunostained endothelial cell separated from adjacent microvessels, tumour cells and other connective tissue elements (Figure 3). Each sample was examined at low power (10 × objective lens and 10 × ocular lens) to identify the area with the highest number of microvessels. The microvessels were then carefully counted in this area on a 250 × field (25 × objective lens and 10 × ocular lens, 0.78 $mm^2$ per field). We considered MC as both continuous and dichotomous variable assuming the median value of 15 vessels as cut-off value to distinguish low from high MC.

### *Vascular endothelial growth factor expression*

Staining for VEGF was performed on 66 out of the entire series (90.4%) using a polyclonal anti-VEGF antibody (Santa Cruz Biotechnology, Santa Cruz, CA, USA) at a 1:50 of dilution overnight. As for the other immunostainings, immunoreaction was displayed using the avidin–biotin peroxidase complex method. The peroxidase activity was visualized with diaminobenzidine. Counterstaining was performed with haematoxylin. Negative controls were carried out by omitting the primary antibody. The VEGF expression was assessed according to the percentage of immunoreactive cells on a total of at least 1000 neoplastic cells. We considered as negative the samples with no immunoreactive cells in their neoplastic component.

### Statistical analyses

All statistical analyses were carried out by the Statistica (Stat-Soft) software system. A chi-square test with Fisher's correction was used to analyse the associations between different variables. The differences between the mean of MC and p53 in patients with or without metastasis and alive or dead were assessed by the unpaired *t*-test as well as VEGF expression in comparison with p53-negative vs p53-positive or low-vascularized vs high-vascularized tumours. The survival analyses were calculated by the Kaplan–Meier method. The differences between tumours with low or high MC and p53 protein were evaluated by the log-rank test.

### RESULTS

The most common histological type was squamous carcinoma (61.6%). Out of 73 tumours 45 (67.1%) were classified as T2 (more than 3 cm in the greatest dimension or invading visceral pleura); 22 out of 73 (30.1%) presented metastatic involvement of

 *© Cancer Research Campaign 1997*

**Table 2** p53 protein expression and MC of 73 cases of NSCLC according to recurrence and overall survival

| Variables | No. of cases | p53 Mean | p53 ±s.d. | p53 *P* | MC Mean | MC ±s.d. | MC *P* |
|---|---|---|---|---|---|---|---|
| Recurrence | | | | | | | |
| No | 40 | 23.5 | 27 | | 15.2 | 9 | |
| | | | | 0.02 | | | 0.0001 |
| Yes | 33 | 38.4 | 29 | | 31.1 | 14 | |
| Overall survival | | | | | | | |
| Alive | 44 | 23.8 | 26 | | 15.7 | 8 | |
| | | | | 0.01 | | | 0.0001 |
| Dead | 29 | 39.9 | 30 | | 32.5 | 14 | |

**Figure 2** Relapse-free (**A**) and overall survival (**B**) in NSCLC patients according to high (> 20) and low (≤ 20) p53 protein expression

**Figure 3** Tumour area with high microvascular count in an invasive NSCLC sample (ABC method; 25 ×)

**Figure 4** Relapse-free (**A**) and overall survival (**B**) of NSCLC patients according to high (> 15) and low (≤ 15) MC

hilar and/or mediastinal lymph nodes and 32 patients developed distant metastases during follow-up. A total of 29 (39.7%) patients died of metastatic disease, while 44 were alive at the moment of analysis.

### p53 expression p53 immunoreactivity was localized in the nuclei of neoplastic cells (Figure 1). The median value (20% of positive cells) was assumed as the cut-off value to distinguish tumours with low or high p53 expression. Tables 1 and 2 report the mean values of p53 protein according to the clinicopathological characteristics and behaviour of the tumours. A higher p53 expression was shown to be associated with late-staged (N1–2; S2–3) and recurring carcinomas ($P = 0.04$; $P = 0.02$). Moreover, patients with high (> 20% of positive cells) p53-expressing tumours showed a significantly shorter relapse-free and overall survival than those with a low (< 20% of positive cells) p53 accumulation in their cancers (Figure 2A: $P = 0.006$; Figure 2B: $P = 0.01$).

**Table 3** Multiple logistic regression to predict overall survival in NSCLC

| Variables | Coefficent b | Standard b error | *t* | *P* |
|---|---|---|---|---|
| Age | 0.1617 | 0.1018 | 1.5875 | 0.1172 |
| Sex | 0.0643 | 0.1059 | 0.6038 | 0.5480 |
| Histotype | 0.1081 | 0.1087 | 0.9943 | 0.3237 |
| N status | 0.2840 | 0.1101 | 2.5789 | 0.0121 |
| Microvessel count | 0.3776 | 0.1085 | 3.4777 | 0.0009 |
| p53 | 0.1141 | 0.1068 | 1.0684 | 0.2892 |

**Table 4** Multiple logistic regression to predict recurrence in NSCLC

| Variables | Coefficent b | Standard b error | *t* | *P* |
|---|---|---|---|---|
| Age | 0.0892 | 0.1020 | 0.8748 | 0.3848 |
| Sex | 0.0089 | 0.1067 | 0.8397 | 0.9333 |
| Histotype | 0.0517 | 0.1089 | 0.4751 | 0.6362 |
| N status | 0.2422 | 0.1103 | 2.1960 | 0.0316 |
| Microvessel count | 0.4336 | 0.1087 | 3.9863 | 0.0001 |
| p53 | 0.1105 | 0.1069 | 1.0336 | 0.3051 |

**Figure 5** Overall survival of NSCLC patients according to high MC and high p53 and all cases except high MC and high p53

### Microvascular count

The area of most intense vascular count was identified for each tumour sample (Figure 3) and the median value of this series (15 vessels per 250 × power field) allowed us to separate tumours with high from tumours with low MC. Tables 1 and 2 show that tumours with metastatic nodal involvement and/or which relapsed during follow-up had a higher number of microvessels compared with cancers with no metastatic involvement ($P = 0.002$; $P = 0.0001$). In addition, shorter relapse-free and overall survival were observed in patients with high microvessel count in their tumours (Figure 4A: $P = 0.00003$; Figure 4B: $P = 0.00001$). Multiple logistic regression analyses reported in Tables 3 and 4 underlined the strong prognostic influence of MC and nodal status on overall ($P = 0.0009$; $P = 0.01$) and relapse-free survival ($P = 0.0001$; $P = 0.03$). The prognostic impact of MC was further confirmed when we analysed the concurrent influence of MC and p53 expression on overall survival. As reported in Figure 5, a significant statistical difference in overall survival was observed between patients whose tumours showed alternatively low MC and low p53, low MC and high p53, high MC and low p53 (condensed below 'All cases except MC > 15p53 > 20') compared with patients whose tumours showed both high MC and high p53 ($P = 0.001$).

**Figure 6** Relation between MC and p53 protein expression in 73 cases of NSCLC (linear regression $r = 0.41$; $P = 0.0003$)

**Table 5** Relationship between MC and p53 protein expression in NSCLC

| Variables | No. of cases | p53 Mean | p53 ± s.d. | *P* |
|---|---|---|---|---|
| Microvessel count | | | | |
| Low | 37 | 19.7 | 22 | 0.001 |
| High | 36 | 41.1 | 31 | |

**Figure 7** Relation between MC and p53 protein in 28 cases of non-squamous subgroup of NSCLC (linear regression $r = 0.58$; $P = 0.001$)

### Relation between p53 expression and microvessel count

Interestingly, when MC was compared with p53 nuclear accumulation, a strong statistical association was found between these two variables (linear regression: $r = 0.41$; $P = 0.0003$) (Figure 6). In fact, mean p53 immunoreactivity was significantly more intense (41.1 ± 31) in tumours with high MC than in those with low MC (19.7 ± 22) ($P = 0.01$), as reported in Table 5. Given the fact that the non-squamous histotype had a higher MC than squamous cell carcinomas, it was interesting to check any correlation between MC and p53 positivity even in the case of histological types. A

© Cancer Research Campaign 1997

**Table 6** VEGF expression according to MC and p53

| Variables | No. of cases | VEGF Mean | VEGF ± s.d. | *P* |
|---|---|---|---|---|
| MC | | | | |
| Low | 34 | 29 | 22 | 0.02 |
| High | 32 | 42.3 | 22 | |
| p53 | | | | |
| Negative | 20 | 25.6 | 21 | 0.02 |
| Positive | 46 | 39.7 | 23 | |
| p53 | | | | |
| Low | 25 | 28 | 20 | 0.04 |
| High | 41 | 40 | 24 | |

**Figure 8** Cytoplasmic VEGF expression in well-differentiated adenocarcinoma of the lung (ABC method; 25 ×)

strong statistical association was found between p53 and MC in non-squamous subtypes as reported in Figure 7 ($r = 0.58$; $P = 0.001$).

### VEGF expression according to MC and p53

VEGF immunoreactivity was detected in the cytoplasm of neoplastic cells with particular accumulation in the perinuclear area (Figure 8) and 63 out of 66 of the tumours (95.4%) expressed VEGF protein (mean 37.1 ± 22; range 5–80%). The median value (40) of the whole series was assumed as the cut-off value to distinguish tumours with low from tumours with high VEGF expression. A significant statistical association was found between VEGF expression and both MC and p53 immunoreactivity. As shown in Table 6, the p53-positive and highly vascularized tumours (MC > 15 vessels per 250 × field) showed a significantly higher VEGF protein expression ($P = 0.02$; $P = 0.02$). The same statistical association was maintained comparing VEGF expression in tumours with low or high p53 according to the median value of p53 (≤ vs > 20% of positive cells) ($P = 0.04$) (Table 6).

### DISCUSSION

In this study, we took into account the role of MC and p53 protein expression in the progression of one of the most frequent and aggressive types of epithelial human cancer, non-small-cell lung carcinoma. Our results have focused on two main aspects: the prognostic impact of MC and p53 expression and the inter-relation between these two factors.

### MC and prognosis

The prognostic role of MC has been widely reported in several types of solid human cancer, such as mammary (Weidner et al, 1991), head and neck (Gasparini et al, 1993), prostate (Weidner et al, 1993), ovarian (Hollingsworth et al, 1995), colorectal (Saclarides et al, 1994), testicular (Olivarez et al, 1994), urotelial (Uaeger et al, 1994), cutaneous (Barnhill et al, 1992), nervous (Li et al, 1994) and bronchial carcinomas (Macchiarini et al, 1992; Yamasaki et al, 1994; Fontanini et al, 1995*a*). These studies, mostly in breast cancers, have shown that intra-tumour microvessel count has an independent prognostic significance when compared with traditional prognostic markers in multivariate analysis (Gasparini and Harris, 1995). In our study, an increasing intra-tumour microvessel count with greater incidence of metastases and/or decreased patient survival has been observed, confirming both ours and other previous data in lung cancer and in other types of solid neoplasms (Macchiarini et al, 1992; Yamasaki et al, 1994; Fontanini et al, 1995*b*; Weidner and Folkman, 1996).

The formation of new capillaries makes it possible for the tumour cells to gain secondary sites successfully and to develop metastases. For this reason, the microvascular bed represents an important requirement for the growth and metastatic spread of primary tumours and also the biological foundation of the prognostic potential of MC.

### p53 expression and prognosis

Alterations of the *p53* gene with consequent nuclear overexpression have been observed in several human cancers, including NSCLC (Lane, 1990), underlining the important role of p53 modifications in tumour development. These alterations seem to be an early event during bronchial cancer progression as demonstrated by several authors who have reported a p53 accumulation in preinvasive lesions of the bronchial tree (Sozzi et al, 1992; Sundaresan et al, 1992; Bennett et al, 1993; Fontanini et al, 1994). However, some experimental evidence has also shown an association between shorter survival and p53 protein expression (Quinlan et al, 1992; Fontanini et al, 1995*a*), although further information will obviously be necessary before this parameter can be used in the prognostic evaluation of human cancers. Indeed, the putative prognostic role of the p53 also observed in this series contrasts with recent results by Lee et al (1995), who report a favourable prognostic influence of p53 expression in a series of 156 resected primary NSCLCs. However, there are substantial differences between ours and Lee et al's methods. As a matter of fact, we observed a lower median p53 value compared with the median value observed by Lee et al (1995) in the histotypes they analysed. The different number of positive cells in their tumours may be caused by the different immunohistochemical procedures, which used microwave oven techniques in formalin-fixed and paraffin-embedded tumour samples, with consequently more consistent staining for the p53 protein. Moreover, the favourable prognostic influence of the p53 overexpression was observed by Lee's group in a subset of NSCLCs with a restricted vision of the p53 prognostic significance. The influence

© Cancer Research Campaign 1997

of the high p53 expression on the outcome of this series of NSCLC patients apparently disagrees with the early expression of the protein during NSCLC development. The early appearance of p53 accumulation is likely to confer to initiated cells a more aggressive phenotype resulting in a faster progression of p53-positive tumours. Thus, the detection of the altered p53 expression may provide prognostic information about the clinical behaviour of primary NSCLC.

### p53 expression and MC

In our study, p53 tumour expression and MC were compared for the analysis of their reciprocal inter-relation. Tumours with high MC both in the entire series and in the non-squamous subgroup presented an increasing p53 expression, suggesting a putative influence of this tumour-suppressor gene on the development of the angiogenic pattern. Recent experimental evidence suggests that new vessel formation in tumours as well as in cultured cell lines is under tumour-suppressor gene control. In a cultured BHK fibroblast cell line converted to anchorage independence and tumorigenicity by loss of a tumour-suppressor gene, Rastinejed et al (1989) have shown that suppressor loss is accompanied by a gain in their ability to acquire the angiogenic phenotype. Moreover, in a glioblastoma cell line that does not express the p53 protein because of an internal rearrangement of the gene and that causes tumours with glioblastoma histology, Van Meier et al (1994) have underlined a down-regulation of angiogenetic capability following introduction of a tetracycline-regulated wild-type *p53* gene into the cells. Concomitantly, in the fibroblast of patients with Li–Fraumeni syndrome, Dameron et al (1995) have demonstrated that the loss of the wild-type p53 is followed by a down-regulation of a potent inhibitor of angiogenesis.

### MC, p53 and VEGF expression

This is the first study in which the relation between MC, p53 and VEGF expressions has been investigated in human lung carcinoma. Recently, Mattern et al (1996) analysed a series of 91 epidermoid lung carcinomas and, apart from the different percentage of cancers expressing the VEGF protein, they found a statistical association between the MC and VEGF expression. They did not analyse the relation between p53 and VEGF, which appears to be strictly connected in the regulation of tumour angiogenesis. Recently, some data have been achieved concerning the influence of wild-type p53 on the human VEGF expression by Mukhopadhyay et al (1995), who demonstrated a pronounced suppressive effect of the VEGF gene expression on an adenovirus-transformed human fetal kidney cell line. On the other hand, a mutant form of p53 has been shown to be implicated in the 12-*O*-tetradecanoylphorbol-13-acetate induction of the VEGF gene expression mediated by the protein kinase C (Kieser et al, 1994). The association between VEGF, MC and p53 underlines on the one hand the important role of VEGF in the control of neoangiogenesis in NSCLC, and on the other hand the hypothesis that the wild-type p53 protein may stop cell cancer development by attracting newly formed vessels.

On the basis of the in vitro and in vivo evidence, we believe that a more detailed analysis of angiogenic growth factors and inhibitor expression in human tumour samples will provide useful information about the genetic control of the angiogenic phenomenon in cancer.

### ABBREVIATIONS

MC, microvessel count; VEGF, vascular endothelial growth factor; NSCLC, non-small-cell lung cancer; OS, overall survival; RFS, relapse-free survival.

### ACKNOWLEDGEMENT

This work has been supported by grants from the Italian Association for Cancer Research (AIRC).

### REFERENCES

Barnhill RL, Fandrey K, Levy MA, Mihm MC and Hyman B (1992) Angiogenesis and tumor progression of melanoma: quantitation of vascularity in melanocytic nevi and cutaneous melanoma. *Lab Invest* **67**: 331–337

Bennett WP, Colby TV, Travis WT, Borkowski A, Jones RT, Lane DP, Metcalf RA, Samet JM, Takeshima Y, Gu JR, Vahakangas KH, Soini Y, Paakko P, Welsh JA, Trump BF and Harris CC (1993) p53 protein accumulates frequently in early bronchial neoplasia. *Cancer Res* **53**: 4817–4822

Dameron KM, Volpert OG, Tainsky MA and Buck N (1994) Control of angiogenesis in fibroblasts by p53 regulation of thrombospondin-1. 1. *Science* **265**: 1582–1584

Fontanini G, Macchiarini P, Pepe S, Ruggiero A, Hardin M, Bigini D, Vignati S, Pingitore R and Angeletti CA (1992) The expression of proliferating cell nuclear antigen in paraffin sections of peripheral node-negative non-small cell lung cancer. *Cancer* **70**: 1520–1527

Fontanini G, Vignati S, Bigini D, Merlo GR, Ribechini A, Angeletti CA, Basolo F, Pingitore R and Bevilacqua G (1994) Human non-small cell lung cancer: p53 protein accumulation is an early event and persists during metastatic progression. *J Pathol* **174**: 23–31

Fontanini G, Bigini D, Vignati S, Basolo F, Mussi A, Lucchi M, Chiné S, Angeletti CA and Bevilacqua G (1995*a*) Microvessel count predicts metastatic disease and survival in non-small cell lung cancer. *J Pathol* **73**: 57–63

Fontanini G, Vignati S, Bigini D, Mussi A, Lucchi M, Angeletti CA, Basolo F and Bevilacqua G (1995*b*) Bcl-2 protein: a prognostic factor inversely correlated to p53 in non-small-cell lung cancer. *Br J Cancer* **72**: 1003–1007

Gasparini G and Harris AL (1995) Clinical importance of the determination of tumor angiogenesis in breast carcinoma: much more than a new prognostic tool. *J Clin Oncol* **13**: 765–782

Gasparini G, Weidner N, Maluta S, Pozza F, Mezzetti M, Testolin A and Bevilacqua P (1993) Intratumoral microvessel density and p53 protein: correlation with metastasis in head-and-neck squamous-cell carcinoma. *Int J Cancer* **55**: 739–744

Harris CC and Hollstein M (1993) Clinical implications of the p53 tumor-suppressor gene. *N Engl J Med* **329**: 1318–1326

Hollingsworth HC, Kohn EC, Steimberg SM, Rothemberg ML and Merino MJ (1995) Tumor angiogenesis in advanced stage ovarian carcinoma. *Am J Pathol* **147**: 33–41

Kieser A, Weich HA, Brandner G, Marmé D and Kolch W (1994) Mutant p53 potentiates protein kinase C induction of vascular endothelial growth factor expression. *Oncogene* **9**: 964–969

Lane DP (1990) Mutations of the p53 gene and accumulation of the p53 protein: common steps found in the majority of human cancers. In *Accomplishments in Cancer Research*. Fortner JG and Rhoads JE (eds) pp. 252–256. Lippincott: Philadelphia

Lee JS, Yoon A, Kalapurakal SK, Ro JY, Lee J, Tu N, Hittelman WN and Hong WK (1995) Expression of p53 oncoprotein in non-small-cell lung cancer: a favorable prognostic factor. *J Clin Oncol* **13**: 1893–1903

Li VW, Folkert RD, Watanabe H, Yu C, Rupnick M, Barnes P, Scott RM, Black PM, Sallan SE and Folkman J (1994) Microvessel count and cerebrospinal fluid basic fibroblast growth factor in children with brain tumors. *Lancet* **334**: 82–86

Macchiarini P, Fontanini G, Hardin JM, Squartini F and Angeletti CA (1992) Relation of neovascularisation to metastasis of non-small-cell lung cancer. *Lancet* **340**: 45–46

Mattern J, Koomagi R and Volm M (1996) Association of vascular endothelial growth factor expression with intratumoral microvessel density and tumor cell proliferation in human epidermoid lung carcinoma. *Br J Cancer* **73**: 931–934

Mountain CF (1986) A new international staging system for lung cancer. *Chest* **89**: 225s–233s

© Cancer Research Campaign 1997

Mukhopadhyay D, Tsiokas L and Sukhatme V (1995) Wild-type P53 and v-Src opposing influence on human vascular endothelial growth factor gene expression. *Cancer Res* **57**: 6161–6165

Olivarez D, Ulbrigh T, Deriese W, Foster R, Reister T, Einhorn L and Sledge G (1994) Neovascularization in clinical stage A testicular germ cell tumor: prediction of metastatic disease. *Cancer Res* **54**: 2800–2802

Quinlan DC, Davidson AS, Summers CL, Warden HE and Doshi HM (1992) Accumulation of p53 correlates with a poor prognosis in human lung cancer. *Cancer Res* **52**: 4828–4831

Rastinejad F, Polverini PJ and Bouck NP (1989) Regulation of the activity of a new inhibitor of angiogenesis by a cancer suppressor gene. *Cell* **56**: 345–355

Saclarides TJ, Speziale NJ, Drab E, Szeluga DJ and Rubin DB (1994) Tumor angiogenesis and rectal carcinoma. *Dis Colon Rectum* **37**: 921–926

Slebos RJC, Kibbelaar RE, Dalesio O, Kooistra A, Stam J, Meijer J, Wagenaar SS, Vanderschueren RGJRA, Van Zandwijk N, Volter JN, Bos JR and Rodenhuis S (1991) K-*ras* oncogene activation as a prognostic marker in adenocarcinoma of the lung. *N Engl J Med* **324**: 1084–1090

Sozzi G, Miozzo M, Denghi R, Pilotti S, Cariani CT, Pastorino U, Della Porta G and Pierotti MA (1992) Deletions of 17p and p53 mutations in preneoplastic lesion of the lung. *Cancer Res* **52**: 6079–6082

Sundaresan V, Ganly P, Hasleton P, Rudd R, Sinha G, Bleehen NM and Rabbitts P (1992) p53 and chromosome 3 abnormalities, characteristic of malignant lung tumours, are detectable in preinvasive lesions of the bronchus. *Oncogene* **7**: 1989–1997

Tateishi M, Ishida T, Mitsudomi T, Kanero S and Sugimachi K (1991) Prognostic value of c-erb-B protein expression in human lung adenocarcinoma and squamous cell carcinoma. *Eur J Cancer* **27**: 1372–1375

Uaeger TM, Weidner N, Chew K, Moore DH, Kerschmann RL, Waldmann RL and Carroll PR (1994) Tumor angiogenesis and lymph node metastases in invasive bladder carcinoma. *J Urol* **151**: 348 (abstract)

Van Meier EG, Polverini PJ, Chazin VR, Su Huang HJ, De Tribolet N and Cavenee WK (1994) Release of an inhibitor of angiogenesis upon induction of wild-type p53 expression in glioblastoma cells. *Nature Genet* **8**: 171–176

Weidner N, Carroll PR, Flax J, Blumenfeld W and Folkman J (1993) Tumor angiogenesis correlates with metastasis in invasive prostate carcinoma. *Am J Pathol* **143**: 401–409

Weidner N, Semple JP, Welch WR and Folkman J (1991) Tumor angiogenesis and metastasis – correlation in invasive breast carcinoma. *N Engl J Med* **324**: 1–8

Weidner N and Folkman J (1996) Tumoral vascularity as a prognostic factor in cancer. In *Important Advances in Oncology*. De Vita VT, Hellmann S, Rosenberg SA (eds) pp. 167–190. Lippincott-Raven: Philadelphia

World Health Organization (1982) The World Health Organization. Histological typing of lung tumours. *Am J Clin Pathol* **77**: 123–136

Yamasaki K, Abe S, Takekawa WM, Sukou N, Watanabe N, Ogura S, Nakajima J, Isobe H, Inoue K and Kawakami JWM (1994) Tumor angiogenesis in human lung adenocarcinoma. *Cancer* **54**: 2245–2250

© Cancer Research Campaign 1997